# Assessment of radiotherapy effect and toxicity using tissue-associated DNA methylation markers in cell-free DNA: a study on prostate cancer

**DOI:** 10.1007/s00411-026-01207-w

**Published:** 2026-03-06

**Authors:** Nurten Bahtiyar, Ozlem Mermut, Sinem Firtina, Ahmet Ozaydin, Aisha Suleymanova, Begum Isikgil, İlhan Onaran

**Affiliations:** 1 Department of Biophysics, Cerrahpaşa Faculty of Medicine, Istanbul University-Cerrahpaşa, Kocamustafapaşa Street, Istanbul, 34098 Turkey; 2 Department of Radiation Oncology, University of Health Sciences, Istanbul Training and Research Hospital, Istanbul, Turkey; 3 Department of Medical Genetics, Cerrahpasa Faculty of Medicine, Istanbul University-Cerrahpasa, Istanbul, Turkey; 4 Department of Medical Biology, Cerrahpasa Faculty of Medicine, Istanbul University-Cerrahpasa, Istanbul, Turkey; 5 Department of Medical Biology and Genetics, Institute for Graduate Education, Istinye University, Istanbul, Turkey

**Keywords:** Prostate cancer, Radiotherapy, Cell-free DNA, Methylation, Variability

## Abstract

This study aimed to evaluate tissue-associated circulating cell-free DNA (cfDNA) as indicators of radiotherapy-related effects and treatment-associated toxicity in prostate cancer. In addition, inter-individual variability in these markers and their responses to radiotherapy-induced cystitis and rectitis were investigated. The DNA methylation status of six tissue-associated genes representing the prostate (*KLK3*,* TGM4*), colon (*MUC2*,* MS4A12*), and bladder (*UPK2*,* DHRS2*) was analyzed in serum-derived cfDNA using methylation-sensitive melting curve analysis (MS-MCA). Five of the six analyzed genes demonstrated significantly higher relative DNA levels in patients with prostate cancer compared with controls. Radiotherapy did not significantly alter the relative DNA levels of these tissue-associated genes. However, cfDNA fragmentation was significantly increased in the post-treatment group compared to the pre-treatment group. Notably, 79-bp DNA fragments were significantly more abundant than 230-bp fragments. In the post-treatment group, no significant correlations were observed between cfDNA fragmentation and the relative DNA levels of most targets, except for an association between the 230-bp fragment and *TGM4* relative DNA levels. These findings suggest limited coupling between cfDNA fragmentation patterns and tissue-associated cfDNA release. Marked inter-individual variability in cfDNA methylation signatures was observed across all examined CpG-containing regions except *UPK2*. This variability did not change significantly following radiotherapy and may therefore limit the utility of these assay regions for monitoring individual radiation-induced tissue damage. Furthermore, no significant changes in methylation profiles of colon- or bladder-associated genes were detected in patients who developed radiotherapy-induced rectitis or cystitis. Overall, increased cfDNA fragmentation following radiotherapy and substantial inter-individual variability in methylation profiles across tissue-associated regions may mask tissue-associated cfDNA signals, thereby complicating the assessment of radiation-induced tissue damage.

**Supplementary Information:**

The online version contains supplementary material available at 10.1007/s00411-026-01207-w.

## Introduction

Prostate cancer is among the most commonly diagnosed malignancies in men, and radiotherapy constitutes a fundamental component of its therapeutic management. One of the major challenges in radiotherapy is the accurate assessment of radiation-induced cellular damage. Another is the determination of individual radiosensitivity in both tumor tissue and surrounding normal tissues, including those not directly irradiated. Although dosimetric studies are widely used to estimate the radiation dose delivered to the target volume and adjacent tissues, investigations focusing on the molecular and cellular effects of radiotherapy at the tissue level remain relatively limited (Li et al. 2016; Barnett et al. 2009). Emerging approaches such as liquid biopsy, advanced immunohistochemical techniques, gene expression-based signatures, and DNA damage response parameters show promise for predicting radiotherapy response and toxicity (Puche-Sanz et al. 2020).

Cancer patients are known to harbor elevated levels of circulating tumor DNA (ctDNA) in their bloodstream. This ctDNA primarily originates from tumor cell death, including apoptosis and necrosis, which can be influenced by factors such as hypoxia, acidic tumor microenvironment conditions, and impaired blood flow (Bittla et al. 2023). Moreover, several studies have demonstrated that cell-free DNA (cfDNA) levels dynamically change during the course of radiotherapy (Cheng et al. 2009; Impey et al. 2016; Hilke et al. 2020). Although cfDNA predominantly consists of short double-stranded DNA fragments of approximately 100–200 base pairs (bp), the presence of much longer fragments, reaching up to ~ 24,000 bp, has also been reported (Oberhofer et al. 2022).

cfDNA molecules are generally considered to retain epigenetic features specific to their cells of origin (Oberhofer et al. 2022; Che et al. 2024). The DNA methylation pattern, which differs between tissue types, arises from differentially methylated blocks containing five or more CpG sites that are unmethylated in one cell type but methylated in other cell types, or vice versa. These tissue-associated methylation signatures enable the identification of the tissue of origin of individual cfDNA fragments and allow estimation of the relative contributions of different tissues or cell types to the circulating cfDNA pool (Oberhofer et al. 2022; Lehmann-Werman et al. 2016). However, some studies have reported inter-individual variability in tissue-associated methylation patterns, which may be influenced by genetic background or unmeasured environmental factors (Titcombe et al. 2022; Planterose Jiménez et al. 2021). Furthermore, the presence of cancer has been shown to modify tissue-associated DNA methylation patterns, raising concerns about the stability of these signatures under pathological conditions (Chen et al. 2016). In parallel, cfDNA fragmentation patterns have been shown to differ among cancer types (Ellinger et al. 2008; Kumari et al. 2022). For instance, an increased proportion of short DNA fragments indicative of apoptotic origin has been observed in patients with prostate cancer (Ellinger et al. 2008). Technical studies have demonstrated that highly fragmented DNA can adversely affect accurate cfDNA quantification, particularly when using quantitative polymerase chain reaction (qPCR) and fluorometric-based measurement methods (Sedlackova et al. 2013; Nakayama et al. 2016). Aberrant de novo methylation of CpG islands is a hallmark of human cancers and can be detected at early stages of carcinogenesis in cancer-related genes, including tumor suppressor genes (Cattrini et al. 2021; Sun et al. 2015). However, it remains unclear whether tissue-associated informative DNA methylation signatures maintain sufficient stability during cancer progression and therapeutic interventions such as radiotherapy.

To address these gaps, the present study aimed to investigate tissue-associated cfDNA methylation signature using methylation-sensitive melting curve analysis (MS-MCA). In addition, the potential of these markers to reflect biological changes associated with the therapeutic effects of prostate radiotherapy and possible treatment-related toxicities was evaluated. Within this framework, changes in the relative DNA levels of tissue-associated genes were analyzed in cfDNA from serum samples collected before and after radiotherapy. These changes were examined in relation to cfDNA levels of apoptosis- and necrosis-associated markers, which are considered indicators of cell death processes. Furthermore, therapy-associated changes in tissue-associated DNA methylation markers and inter-individual variability were evaluated, and the potential relationships between this variability and radiotherapy-induced cystitis and rectitis were investigated.

## Materials and methods

### Case selection

This study included a total of 52 male participants: 25 patients diagnosed with prostate adenocarcinoma who were treated with radiotherapy (mean age 66.33 years; range 46–82 years) and 27 healthy male individuals who constituted the control group (mean age 63.36 years; range 53–76 years). For patients with prostate cancer, exclusion criteria included a history of distant metastasis or other malignant diseases, diabetes mellitus, hypertension, chronic inflammatory diseases, infectious diseases, and autoimmune disorders. Radiotherapy was administered using a device capable of volumetric modulated arc therapy (VMAT) (Varian Trilogy). Patients received daily fractions of 200 cGy, five days per week, for approximately eight weeks, resulting in a total dose of 78 Gy. The study protocol was reviewed and approved by the Ethics Committee of the Cerrahpaşa Medical Faculty, Istanbul University-Cerrahpaşa (approval number: A-39). The study was conducted in accordance with the Declaration of Helsinki and its subsequent amendments. Written informed consent was obtained from all participants prior to enrollment in the study.

### Sample collection

Peripheral venous blood samples (5 mL) were collected from all participants using serum separator tubes without anticoagulants. For patients with prostate cancer, blood samples were collected at two predefined time points: (i) one day prior to the initiation of radiotherapy (Pre-RT group) and (ii) on the final day of radiotherapy following completion of the prescribed treatment course (Post-RT group). Healthy control participants provided a single peripheral blood sample of the same volume for analysis. After collection, samples were allowed to clot at room temperature for approximately 30 min and were subsequently centrifuged at 3,000 rpm for 10 min to separate the serum. The serum fraction was carefully transferred into sterile microcentrifuge tubes and stored at − 80 °C until cfDNA extraction.

### Determination of relative levels of mtDNA-79 and mtDNA-230

Circulating DNA was extracted from serum samples using the Plasma/Serum Cell-Free Circulating DNA Purification Kit (Norgen Biotek, Canada) according to the manufacturer’s instructions. qPCR was employed to assess mitochondrial DNA (mtDNA) fragments in serum. Two primer sets targeting the mitochondrial 16S rRNA gene were used to amplify a 79-bp fragment (mtDNA-79) and a 230-bp fragment (mtDNA-230). The forward primer for both fragments was 5’-CAGCCGCTATTAAAGGTTCG-3’, while the reverse primers were 5’-CCTGGATTACTCCGGTCTGA-3’ for mtDNA-79 and 5’-GGGCTCTGCCATCTTAACAA-3’ for mtDNA-230. qPCR reactions were performed in duplicate using a Real-Time PCR Detection System (Bio-Rad Laboratories, Hercules, CA, USA). Each 20 µL reaction mixture comprised 2 µL of DNA, 10 µL of BlasTaq™ 2X qPCR MasterMix (Abm, Canada), and 0.40 µL of each forward and reverse primer (10 mM). PCR conditions were 95 °C for 3 min, followed by 40 cycles at 95 °C for 15 s, 60 °C for 60 s, and 72 °C for 30 s. Melting curve analysis (MCA) was performed to verify the specificity of the PCR products. The housekeeping gene β-actin was used to normalize qPCR data. The forward primer for β-actin was 5’-AGGATGCAGAAGGAGATCACTG-3’ and the reverse primer was 5’-GGGTGTAACGCAACTAAGTCATAG-3’. Relative mtDNA levels were calculated using the 2^−ΔCt^ method and normalized to β-actin. mtDNA integrity, reflecting the degree of fragmentation, was determined as the ratio of relative mtDNA-230 to mtDNA-79 levels.

### Identification of tissue-associated cfDNA methylation markers

In this study, healthy tissue data obtained from The Cancer Genome Atlas (TCGA) Program (10.1038/ng.2764), the Human Protein Atlas (https://www.proteinatlas.org/), the Tissue-Specific Gene Expression and Regulation (TiGER) database (http://bioinfo.wilmer.jhu.edu/tiger/), and the literature (Loyfer et al. 2023) were comparatively analyzed using an integrative approach. Based on these analyses, tissue-associated unmethylated markers were identified. *Kallikrein-related peptidase 3 (KLK3)* and *transglutaminase 4 (TGM4)* genes were selected to assess the methylation status associated with prostate tissue. Similarly, *mucin 2 (MUC2)* and *membrane-spanning 4-domains subfamily A member 12 (MS4A12)* genes were used to evaluate the methylation status associated with colon tissue, whereas *dehydrogenase/reductase 2 (DHRS2)* and *uroplakin 2 (UPK2*) genes were chosen to represent the methylation status of bladder-derived cfDNA.

### Evaluation of tissue-associated gene methylation status and relative DNA levels

The methylation status of CpG islands in six selected genes was analyzed in circulating cfDNA. Total DNA was isolated from serum samples using the Quick-DNA™ Miniprep Kit (Zymo Research, Cat. No. D3024) according to the manufacturer’s protocol. Bisulfite conversion of the extracted DNA was performed using the EZ DNA Methylation Kit (Zymo Research Corporation, Cat. No. D5001) following the manufacturer’s instructions. Subsequently, bisulfite-treated DNA was subjected to MS-MCA using the ABI OneStepPlus Real-Time PCR Detection System (Applied Biosystems, USA). Each 20 µL PCR reaction contained 2 µL of bisulfite-converted DNA, 10 µL of BlasTaq™ 2X qPCR MasterMix (Abm, Canada), and 0.40 µL of each forward and reverse primer (10 mM). PCR amplification was carried out under the following conditions: initial denaturation at 95 °C for 10 min, followed by 40 cycles of denaturation at 95 °C for 15 s, annealing at 60 °C for 60 s, and extension at 72 °C for 30 s. Primer sequences used for MS-MCA are listed in Supplementary Table 1. DNA methylation status was determined based on differences in melting temperatures between methylated and unmethylated DNA following bisulfite treatment. After amplification, MCA of the PCR products was performed. Bisulfite conversion preserves CG dinucleotides in methylated DNA, whereas unmethylated cytosines are converted to thymine, resulting in CG-to-TG transitions and a reduced melting temperature. Accordingly, higher Tm values indicated CG-rich, methylated DNA, while lower Tm values corresponded to TG-converted, unmethylated DNA. Relative DNA levels were calculated using the 2^−ΔCt^ method and normalized to the β-actin reference gene.

### Statistical analysis

Continuous variables are presented as mean ± standard deviation (SD), while categorical variables are expressed as counts. The methylation ratio for each gene and each sample was calculated as the ratio of methylated cytosines (mC) to total cytosines (mC + C) across all analyzed CpG sites and expressed as a percentage. Samples with DNA methylation levels exceeding the mean plus two standard deviations of the normal control samples were classified as hypermethylated. Samples with methylation levels within the mean ± two standard deviations of the normal controls were defined as normal-like methylated. In contrast, samples with DNA methylation levels below the mean minus two standard deviations of the normal controls were considered hypomethylated (Klajic et al. 2013). Statistical analyses were performed using SPSS software version 22.0 (SPSS Inc., Chicago, IL, USA) and GraphPad Prism version 5.0 (GraphPad Software, San Diego, CA). Data normality was assessed using the Shapiro-Wilk test. As most variables did not follow a Gaussian distribution, non-parametric statistical tests were applied. Differences in continuous variables between groups were evaluated using the Mann-Whitney U test or the Wilcoxon test, as appropriate. Differences in categorical variables were analyzed using Fisher’s exact test. Spearman’s rank correlation coefficient was used to assess associations between quantitative variables. A p value < 0.05 was considered statistically significant.

## Results

### Relative mtDNA-79 and mtDNA-230 levels in serum samples

**Fig. 1:**
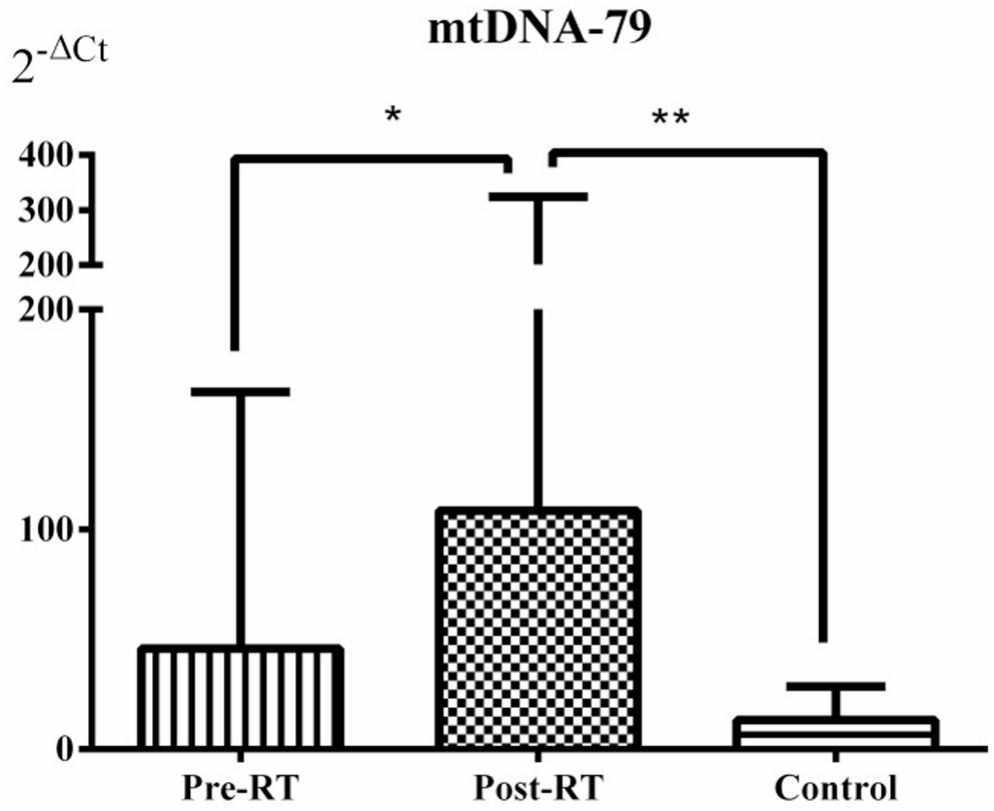
Serum mtDNA-79 relative levels in the patient and control groups. Values are expressed as the mean ± standard deviation. Data were analyzed with Mann-Whitney U and Wilcoxon tests. *p = 0.021, **p = 0.007

**Fig. 2:**
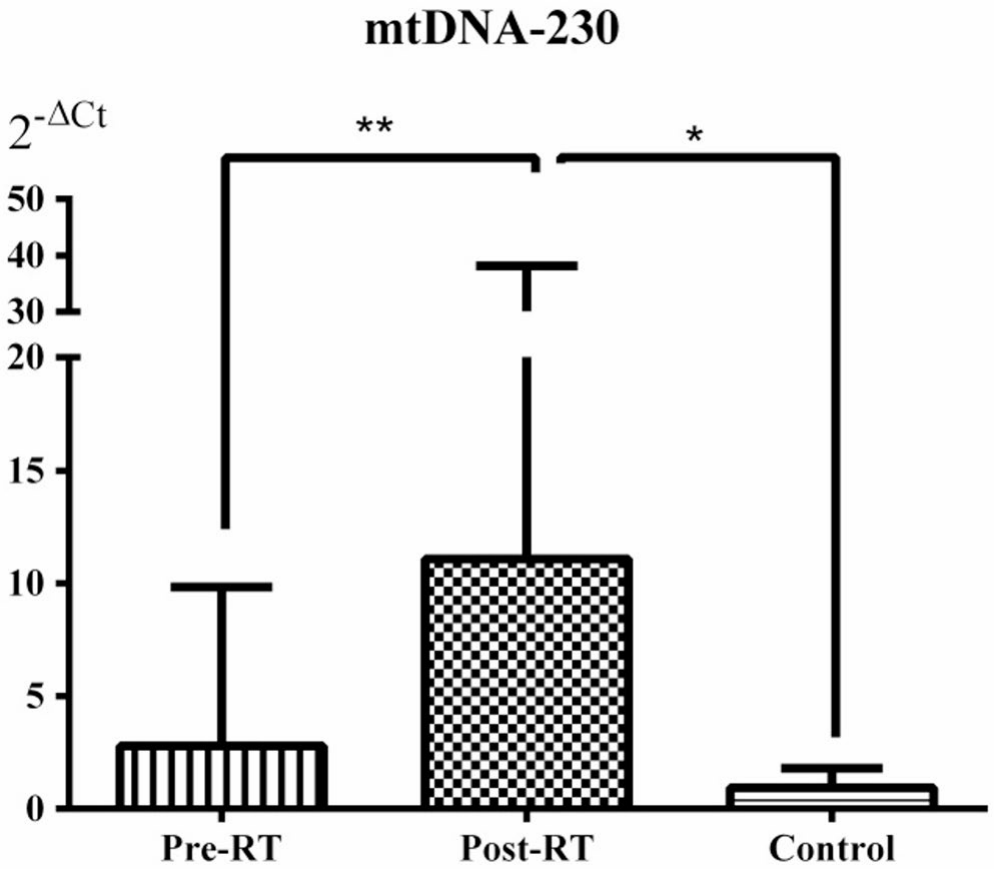
Serum mtDNA-230 relative levels in the patient and control groups. Values are expressed as the mean ± standard deviation. Data were analyzed with Mann-Whitney U and Wilcoxon tests. *p = 0.029, **p = 0.002

The relative DNA levels (2^−ΔCt^) of mtDNA-79 and mtDNA-230 were significantly increased in the Post-RT group compared with the Pre-RT group (*p* = 0.021 and *p* = 0.002, respectively). Similarly, the relative DNA levels of mtDNA-79 and mtDNA-230 were significantly higher in the Post-RT group than in the Control group (*p* = 0.007 and *p* = 0.029, respectively). Although serum mtDNA-79 and mtDNA-230 levels in the Pre-RT group were higher than those in the Control group, these differences did not reach statistical significance (both *p* > 0.05) (Figs. 1 and 2).

**Fig. 3:**
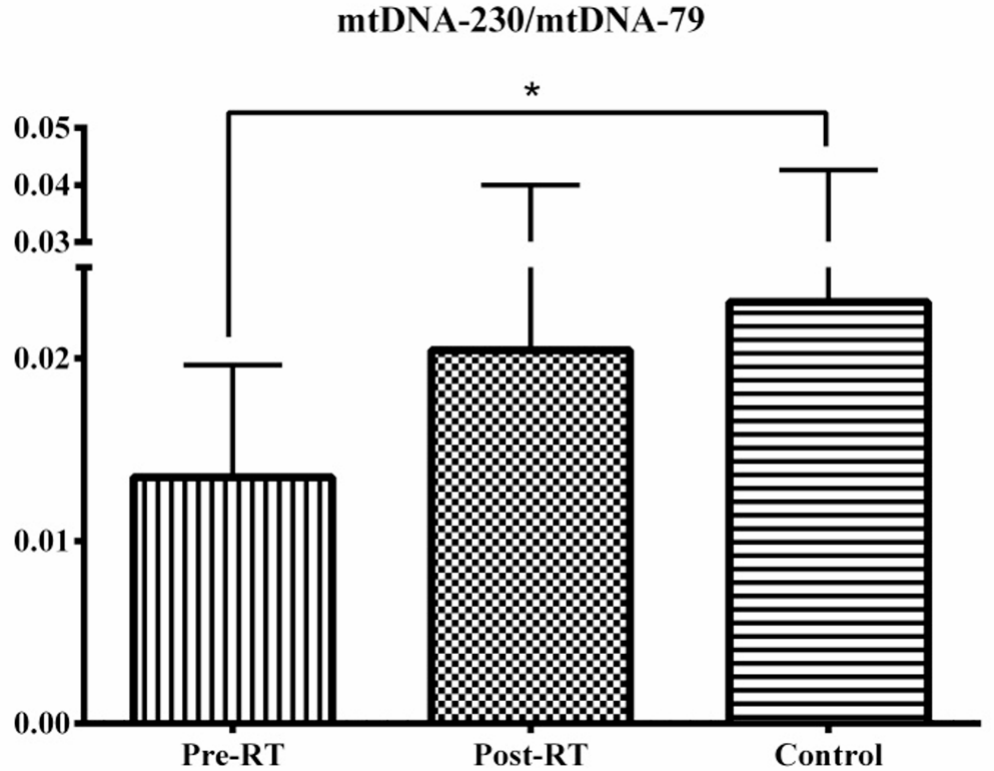
Serum mtDNA Integrity levels in the patient and control groups. Values are expressed as the mean ± standard deviation. Data were analyzed with Mann-Whitney U and Wilcoxon tests. *p = 0.036

mtDNA integrity, defined as the ratio of long to short mtDNA fragments, was evaluated in the Control, Pre-RT, and Post-RT groups. A significant difference was observed between the Pre-RT and Control groups (*p* = 0.036), with the highest mtDNA integrity observed in the Control group. No significant differences were observed between the remaining groups (Fig. 3).

### Relative DNA levels of selected genes in serum samples

**Fig. 4:**
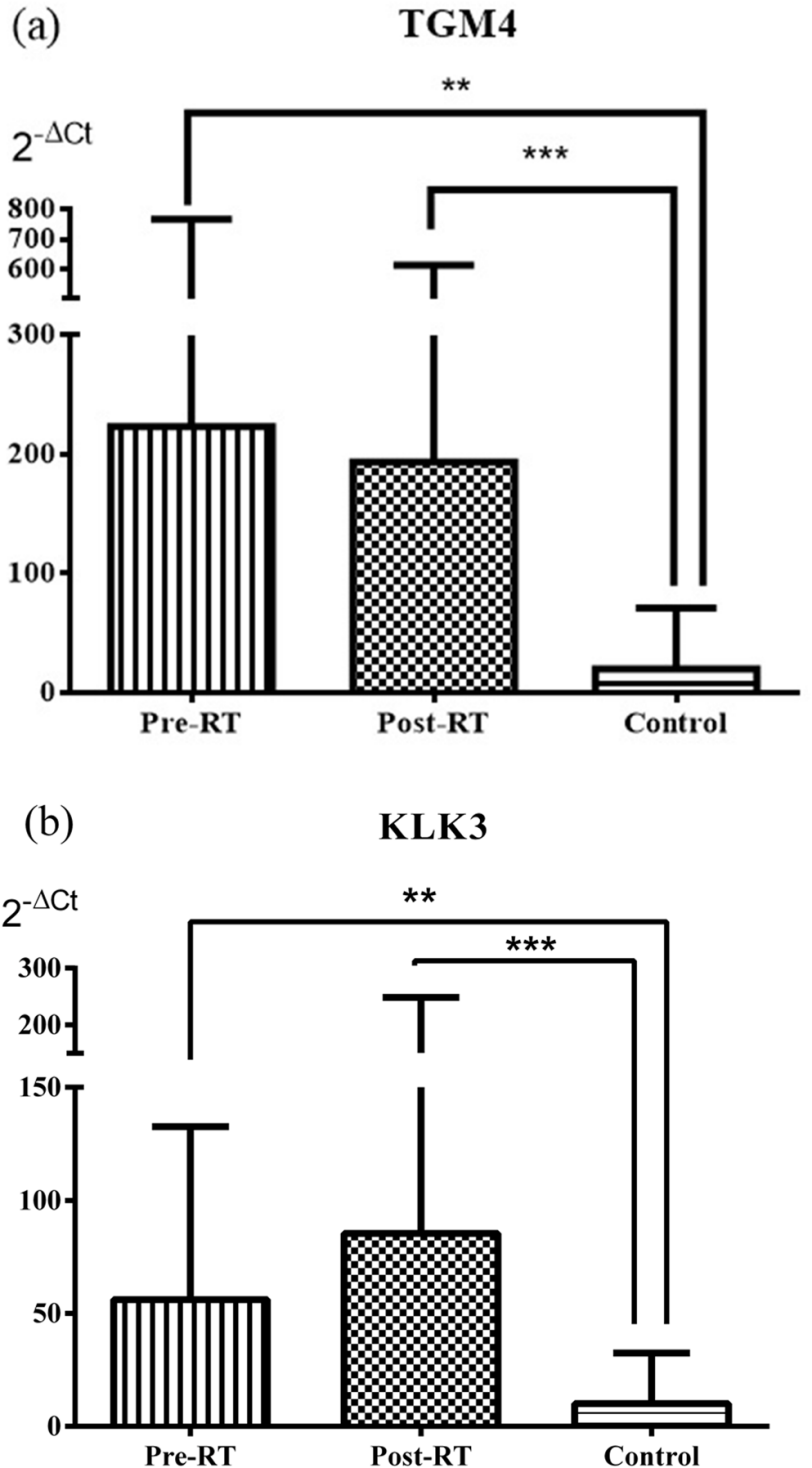
Relative DNA levels of the prostate-associated genes *TGM4* and *KLK3* in serum from patient and control groups, expressed as mean ± standard deviation (**a**) for *TGM4* and (**b**) for *KLK3*. Data were analyzed with Mann-Whitney U and Wilcoxon tests. *TGM4* (**p = 0.003, ***p < 0.001), and *KLK3* (**p = 0.002, ***p < 0.001)

**Fig. 5:**
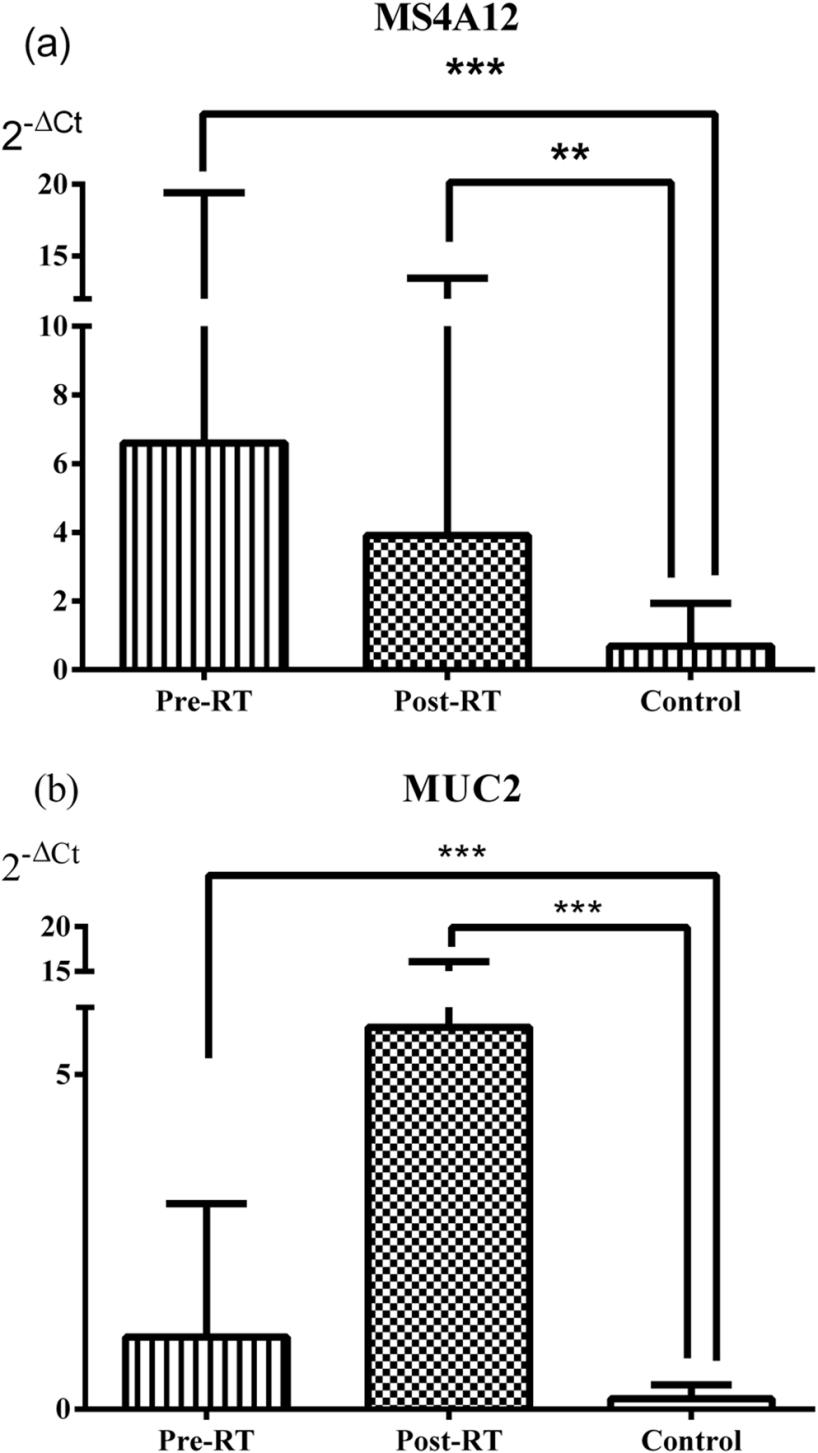
Relative DNA levels of the colon-associated genes *MS4A12* and *MUC2* in serum from patient and control groups, expressed as mean ± standard deviation (**a**) for *MS4A12* and (**b**) for *MUC2*. Data were analyzed with Mann-Whitney U and Wilcoxon tests. *MS4A12* (**p = 0.008, ***p < 0.001), and *MUC2* (***p < 0.001, for both)

**Fig. 6:**
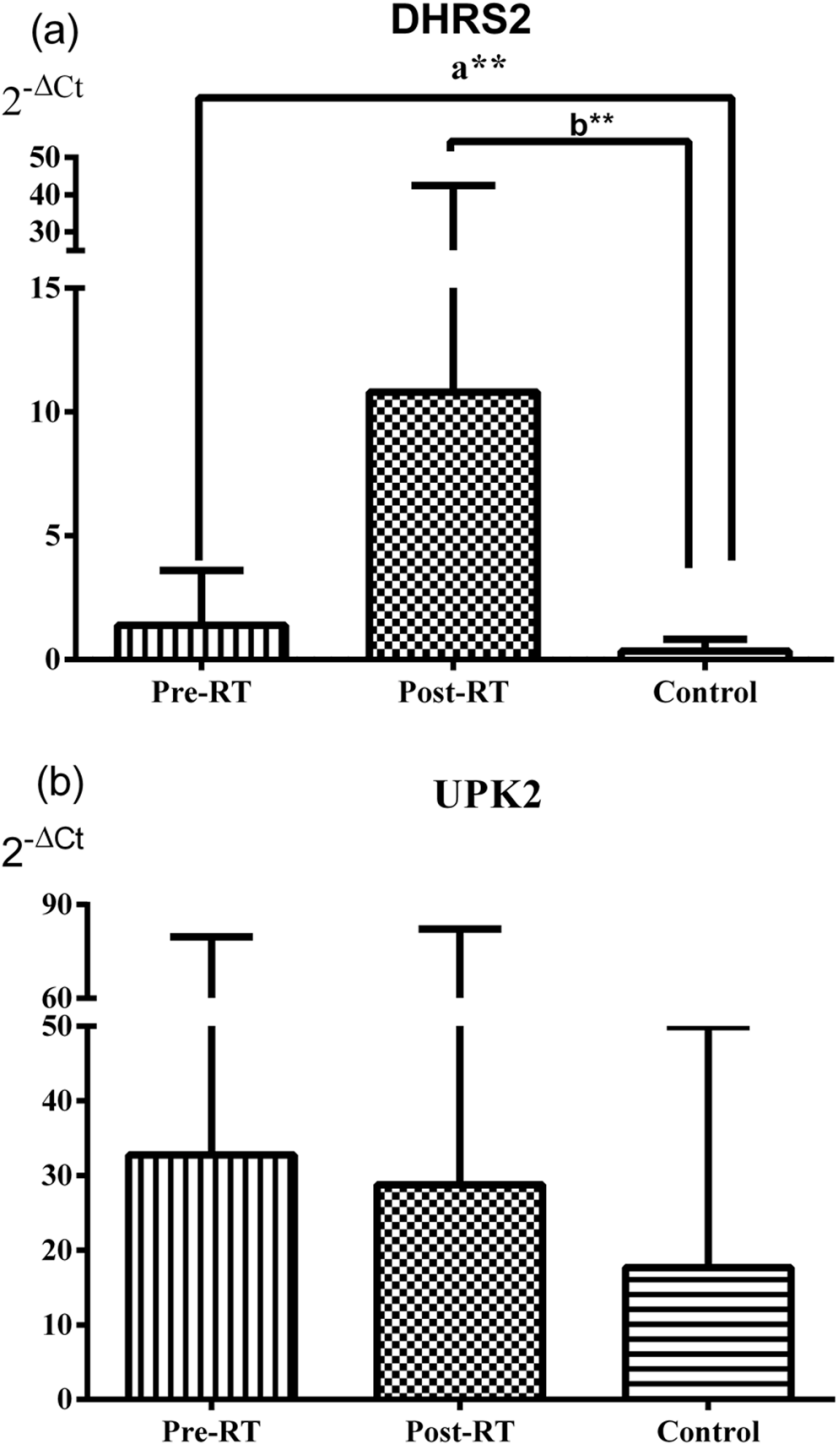
Relative DNA levels of the bladder-associated genes *DHRS2* and *UPK2* in serum from patient and control groups, expressed as mean ± standard deviation (**a**) for *DHRS2* and (**b**) for *UPK2*. Data were analyzed with Mann-Whitney U and Wilcoxon tests. *DHRS2* (a**p = 0.003, b**p = 0.008), *UPK2* (*p* > 0.05, for all)

The relative DNA levels (2^-ΔCt^) of the *TGM4* were significantly increased in both the Pre-RT and Post-RT groups compared with the Control group (*p* = 0.003 and *p* < 0.001, respectively). Similarly, the relative DNA levels of the *KLK3* were significantly higher in the Pre-RT and Post-RT groups than in the Control group (*p* = 0.002 and *p* < 0.001, respectively) (Fig. 4). The relative DNA levels of the *MS4A12* were significantly increased in the Pre-RT and Post-RT groups compared with the Control group (*p* < 0.001 and *p* = 0.008, respectively). Likewise, the values of the *MUC2* were significantly higher in both the Pre-RT and Post-RT groups than in the Control group (*p* < 0.001 for both) (Fig. 5). The relative DNA levels of the *DHRS2* were significantly increased in the Pre-RT and Post-RT groups compared with the Control group (*p* = 0.003 and *p* = 0.008, respectively). In contrast, no significant differences were observed among the groups in the 2^-ΔCt^ values of the *UPK2* (all *p* > 0.05) (Fig. 6).

### Relative DNA levels in patients with and without cystitis or rectitis

**Fig. 7:**
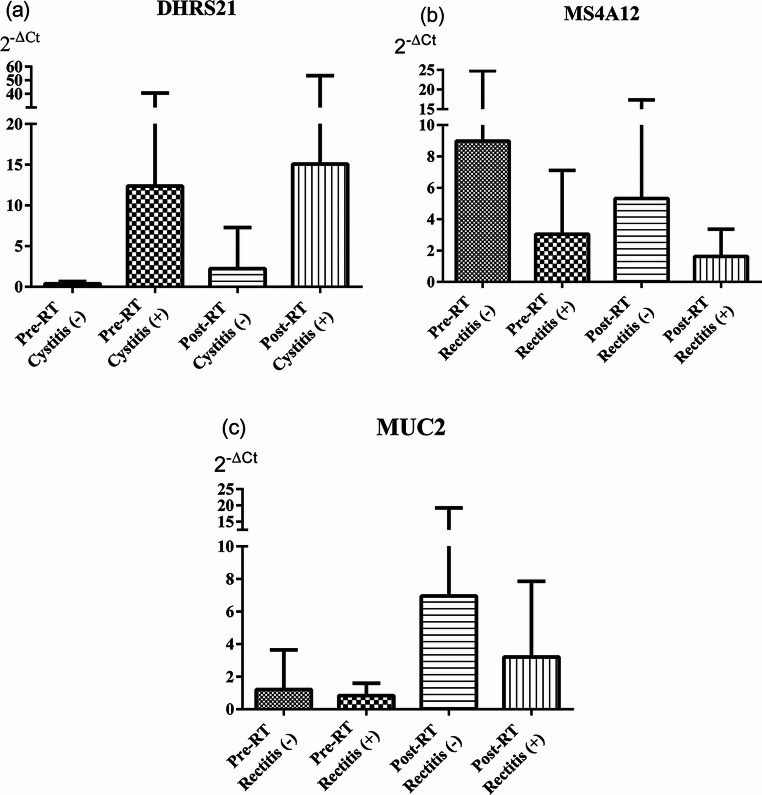
Relationship of cystitis and rectitis development with serum relative DNA levels in the patient groups: (**a**) *DHRS2*, (**b**) *MS4A12*, and (**c**) *MUC2*. Data are expressed as mean ± standard deviation and analyzed using the Wilcoxon test

The association between radiotherapy-induced cystitis and relative DNA levels of *DHRS2* was analyzed. *DHRS2* (2^−∆Ct^) values tended to increase in both patient groups, with and without cystitis, although the differences were not statistically significant. Similarly, the relationship between rectitis and the relative DNA levels of *MS4A12* and *MUC2* was evaluated. Following treatment, *MS4A12* (2^−∆Ct^) values decreased, whereas *MUC2* (2^−∆Ct^) values increased in both patient groups, but these changes did not reach statistical significance (Fig. 7).

### Methylation profiles of tissue-associated cfDNA markers

When the methylation profile of prostate tissue-associated genes were examined, *TGM4* was found to exhibit multiple methylation profiles across all groups. Six distinct methylation profiles were identified in both the Pre-RT and Post-RT groups, whereas the number of profiles in the Control group was limited to four. Across all groups, 100% unmethylated (100%U) and 100% hemimethylated (100%H) profiles were predominant, while partially methylated profiles were observed at lower frequencies. In contrast, only two methylation profiles (100%U and 100%H) were detected for the *KLK3* gene in all groups, with no notable variation in profile distribution among the groups (Supplementary Table 2).

When the methylation profiles of genes associated with colon tissue were examined, a small number of partially methylated profiles were detected in the *MS4A12* gene in the Pre-RT group, while only 100%U and 100%H profiles were determined in the Post-RT and Control groups. The predominant profile across all groups was 100%H. Similarly, only two methylation profiles (100%U and 100%H) were identified for *MUC2*, with comparable distributions among the groups (Supplementary Table 3).

Evaluation of bladder tissue–associated genes revealed greater methylation heterogeneity in *DHRS2* compared with the other genes analyzed. Six and seven distinct methylation profiles were identified in the Pre-RT and Post-RT groups, respectively, whereas four profiles were observed in the Control group. In contrast, *UPK2* was 100%U across all study groups (Supplementary Table 4).

### DNA methylation status of selected genes

The *TGM4* gene exhibited three different methylation statuses (hypermethylated, hypomethylated, and normal-like methylated) in the Pre-RT, Post-RT, and Control groups; however, no statistically significant differences in methylation statuses were observed among the groups (*p* = 0.453). Similarly, analysis of the *KLK3* gene revealed two different methylation statuses (hypomethylated and normal-like methylated) in the Pre-RT, Post-RT, and Control groups, with no significant differences in methylation distribution detected among the groups (*p* = 0.378) (Table 1).

**Table 1:** Evaluation of methylation status in the patient and control groups

Gene	Pre-RT (*n*=25)	Post-RT (*n*=25)	Control (*n*=27)	p
***TGM4***				
Methylation status				
Hypermethylated Hypomethylated Normal-like methylated	2 14 9	- 18 7	- 18 9	p=0.453
***KLK3***				
Methylation situation				
Hypomethylated Normal-like methylated	9 16	12 13	15 12	p=0.378
***MS4A12***				
Methylation situation				
Hypomethylated Normal-like methylated	2 23	4 21	13 14	p=0.001
***MUC2***				
Methylation situation				
Hypomethylated Hypermethylated	14 11	18 7	19 8	0.474
***DHRS2***				
Methylation situation				
Hypomethylated Normal-like methylated	8 17	16 9	12 15	p=0.081

The evaluation was made according to the of methylation status. Data were analyzed using the Fisher’s Exact test

The *MS4A12* gene also exhibited two different methylation statuses (hypomethylated and normal-like methylated) in the Pre-RT, Post-RT, and Control groups. The number of cases with hypomethylated statuses was significantly lower in the Pre-RT and Post-RT groups compared with the Control group, and a statistically significant difference in methylation distribution was observed among the groups (*p* = 0.001). The *MUC2* gene showed two methylation statuses (hypomethylated and hypermethylated) across all groups; however, no significant differences in methylation statuses were observed between the groups (*p* = 0.474).

The *DHRS2* gene exhibited two methylation statuses (hypomethylated and normal-like methylated) in the Pre-RT, Post-RT, and Control groups, with no statistically significant differences in methylation statuses among the groups (*p* = 0.081). The *UPK2* gene was 100%U in all groups, with no methylation changes detected (Table 1).

### Methylation changes in selected genes following radiotherapy

Table 2 summarizes the methylation changes observed in selected genes following radiotherapy in prostate cancer patients. The *TGM4* gene showed both increases and decreases in methylation status between the Pre-RT and Post-RT groups, although a subset of patients exhibited no change in methylation status after treatment. Similarly, the *KLK3* gene demonstrated bidirectional transitions between hypomethylated and normal-like methylated statuses following radiotherapy, while the methylation status remained unchanged in some patients.

**Table 2:** Methylation change with radiotherapy in prostate cancer patients

	Pre-RT (n=25)	Post-RT (n=25)	n
**TGM4**	Hypermethylated Hypomethylated Normal-like methylated Hypomethylated Normal-like methylated	Hypomethylated Normal-like methylated Hypomethylated Hypomethylated Normal-like methylated	2 5 7 9 2
***KLK3***	Hypomethylated Normal-like methylated Normal-like methylated Hypomethylated	Normal-like methylated Hypomethylated Normal-like methylated Hypomethylated	5 8 8 4
***MS4A12***	Hypomethylated Normal-like methylated Normal-like methylated Hypomethylated	Normal-like methylated Hypomethylated Normal-like methylated Hypomethylated	1 3 20 1
***MUC2***	Hypomethylated Hypermethylated Hypermethylated Hypomethylated	Hypermethylated Hypomethylated Hypermethylated Hypomethylated	6 11 1 7
***DHRS2***	Hypomethylated Normal-like methylated Normal-like methylated Hypomethylated	Normal-like methylated Hypomethylated Normal-like methylated Hypomethylated	1 7 10 7

Classification was performed based on the methylation status

For the *MS4A12* gene, the methylation status remained unchanged in the majority of cases, with only a small number of patients exhibiting alterations between hypomethylated and normal-like methylated statuses after treatment. The *MUC2* gene showed relatively frequent methylation changes following radiotherapy, with transitions observed between hypomethylated and hypermethylated states; however, the methylation status remained stable in a subset of patients.

Likewise, the methylation status of the *DHRS2* gene remained unchanged in most patients, although bidirectional changes between hypomethylated and normal-like methylated statuses were observed in a limited number of cases (Table 2).

### Methylation changes in relation to radiotherapy-induced cystitis and rectitis

Table 3 presents the methylation changes associated with the development of radiotherapy-induced cystitis and rectitis in patients with prostate cancer. Among the genes evaluated in relation to cystitis development, *DHRS2* exhibited post-radiotherapy methylation changes in both the Cystitis (+) and Cystitis (−) groups. In a subset of Cystitis (+) patients, *DHRS2* shifted from normal-like methylation to hypomethylation, and similar changes were also observed in Cystitis (−) patients. In some patients, the methylation status remained unchanged following radiotherapy. However, no statistically significant difference in methylation changes was observed between the groups (*p* = 0.150). The *UPK2* gene remained fully hypomethylated in all patients and showed no association with the development of cystitis.

**Table 3:** Relationship of Cystitis and Rectitis Development with Methylation Status in the Pre- and Post-RT Groups

	Pre-RT	Post-RT	n
**DHRS21**			
**Cystitis (-) (n=8)**	Normal-like methylated	Normal-like methylated	4
	Normal-like methylated	Hypomethylated	3
	Hypomethylated	Hypomethylated	1
	Hypomethylated	Normal-like methylated	0
**Cystitis (+) (n=17)**	Normal-like methylated	Normal-like methylated	5
	Normal-like methylated	Hypomethylated	5
	Hypomethylated	Hypomethylated	6
	Hypomethylated	Normal-like methylated	1
**MS4A12**			
**Rectitis (-) (n=18)**	Normal-like methylated	Normal-like methylated	15
	Normal-like methylated	Hypomethylated	2
	Hypomethylated	Normal-like methylated	0
	Hypomethylated	Hypomethylated	1
**Rectitis (+) (n=7)**	Normal-like methylated	Normal-like methylated	5
	Normal-like methylated	Hypomethylated	1
	Hypomethylated	Normal-like methylated	1
	Hypomethylated	Hypomethylated	0
**MUC2**			
**Rectitis (-) (n=17)**			
	Hypermethylated	Hypermethylated	1
	Hypermethylated	Hypomethylated	8
	Hypomethylated	Hypermethylated	4
	Hypomethylated	Hypomethylated	4
**Rectitis (+) (n=8)**	Hypermethylated	Hypermethylated	0
	Hypermethylated	Hypomethylated	3
	Hypomethylated	Hypermethylated	1
	Hypomethylated	Hypomethylated	4

Classification was performed based on the methylation status

Regarding rectitis development, *MS4A12* showed methylation changes in a limited number of patients in both the Rectitis (+) and Rectitis (−) groups, while the majority of patients exhibited no post-radiotherapy changes. No significant difference was observed between the groups (*p* = 0.644). For the *MUC2* gene, both the Rectitis (+) and Rectitis (−) groups included patients who transitioned from hypomethylated to hypermethylated states or vice versa following radiotherapy, whereas others maintained an unchanged methylation status. Similarly, no statistically significant association was found between *MUC2* methylation changes and the development of rectitis (*p* = 0.235) (Table 3).

### Correlation analysis of mtDNA fragmentation, mtDNA integrity, tissue-associated relative DNA markers, and methylation ratios in pre- and post-radiotherapy groups

In the Pre-RT group, mtDNA integrity showed positive correlations with the relative DNA levels of mtDNA-230, *TGM4*,* KLK3*,* MS4A12*,* MUC2*,* DHRS2*, and *UPK2.* Positive correlations were also observed between the relative DNA levels of these genes, including mtDNA-230 with *TGM4*,* KLK3*,* and MS4A12*; *TGM4* with *MS4A12*,* MUC2*, and *DHRS2*; *KLK3* with *MS4A12*,* MUC2*,* DHRS2*, and *UPK2*; and *MS4A12* with *MUC2*,* DHRS2*, and *UPK2*. Additionally, the methylation ratios of *MUC2* and *DHRS2* were positively correlated. In contrast, mtDNA integrity was negatively associated with mtDNA-79, and *KLK3* relative DNA levels showed a negative correlation with *TGM4* methylation ratios (Supplementary Table 5).

In the Post-RT group, several significant positive correlations were identified among the relative DNA levels of the analyzed markers. The relative DNA level of mtDNA-230 was positively correlated with that of *TGM4*. Also, *TGM4* levels showed positive correlations with the relative DNA levels of *MS4A12*, *MUC2*, and *DHRS2*. Relative DNA levels of *KLK3* were positively correlated with those of *MS4A12*, *MUC2*, *DHRS2*, and *UPK2*, as well as with the methylation ratio of *MS4A12*. Additionally, *MS4A12* relative DNA levels were positively associated with the levels of *MUC2* and *DHRS2* and with its own methylation ratio. Furthermore, the relative DNA level of *MUC2* was positively correlated with those of *DHRS2* and *UPK2*, while *DHRS2* relative DNA levels were positively associated with *UPK2* levels. Finally, a significant positive correlation was observed between the methylation ratios of *MUC2* and *DHRS2* (Supplementary Table 6).

## Discussion

Methylation-based assays have emerged as promising tools for the early detection of disease, as alterations in DNA methylation patterns can serve as indicators of tissue- or organ-specific damage. Such epigenetic changes have been successfully utilized to monitor tissue cell death across a broad spectrum of human pathologies (Lehmann-Werman et al. 2016; Lo et al. 2021). Among currently available approaches, MS-MCA represents a widely applied method for DNA methylation analysis, offering notable advantages over pyrosequencing, next-generation sequencing, and microarray-based platforms in terms of cost-effectiveness and suitability for high-throughput sample processing. In addition, MS-MCA allows objective characterization and quantification of DNA methylation in biological specimens, thereby facilitating robust statistical comparisons across samples.

A key component of methylation-based cfDNA analysis involves estimating the relative contribution of DNA released from specific tissues into the circulating cfDNA pool. This process requires careful selection of genomic target regions and optimized primer design for PCR-based assays. In the present study, relative quantification of six tissue-associated genes demonstrated that five genes exhibited elevated relative DNA levels in the serum cfDNA of patients with localized prostate cancer compared with healthy controls. These findings support the potential utility of methylation-based cfDNA analysis for detecting disease-associated tissue damage.

Given that DNA fragmentation increased markedly following radiotherapy, we initially hypothesized that the relative DNA levels of prostate-associated tissue genes would also increase after treatment. Contrary to this expectation, relative DNA levels of these genes did not change significantly. This observation suggests that relying solely on the relative abundance of tissue-associated DNA markers may be insufficient for accurately assessing radiation-induced cellular damage or for evaluating the individual radiosensitivity of tumor tissue and surrounding normal tissues.

Based on existing literature and our findings, two major factors may explain the absence of increased prostate-associated gene DNA levels despite elevated cfDNA fragmentation: the increased abundance of short DNA fragments and inter-individual variability in DNA methylation within tissue-associated differentially methylated or unmethylated regions. Increased mitochondrial damage during radiotherapy may enhance apoptosis and vacuolization, leading to increased release of short DNA fragments. Accordingly, quantitative assessment of apoptotic 79-bp fragments in serum may provide a more sensitive indicator of radiotherapy-associated toxicity.

Importantly, cfDNA fragmentation does not occur uniformly across the genome; instead, certain genomic regions may be overrepresented while others are underrepresented within circulating DNA fragments (Yeh 2020; Beck et al. 2009). In fragmented DNA samples, PCR-based assays quantify intact genomic regions of a defined amplicon length. Consequently, the highly fragmented nature of cfDNA and reduced median fragment size may compromise primer binding efficiency and reduce the accuracy of methylation-specific PCR assays (Sedlackova et al. 2013; Nakayama et al. 2016). Consistent with this concept, the apoptotic 79-bp fragment was more abundant than the necrotic 230-bp fragment in the serum of localized prostate cancer patients and showed greater fragmentation compared with healthy controls. Fragment levels further increased after treatment, yet these changes were independent of the relative DNA abundance of selected tissue-associated gene promoter regions (Ellinger et al. 2008, 2012). These findings suggest that the expected linear positive association between treatment-related toxicity and increased relative DNA levels of tissue-associated genes may be disrupted by elevated fragmentation. Increased short fragments may reduce primer binding probability to target regions, leading to apparently reduced concentrations in PCR-based measurements (Sedlackova et al. 2013). Therefore, additional methodological refinements are needed to improve the evaluation of tissue-associated DNA levels through epigenetic signatures, particularly in cfDNA samples enriched with short fragments. Another important factor influencing analytical performance is inter-individual variability in DNA methylation at tissue-associated genes (Planterose Jiménez et al. 2021). Previous studies have shown that methylation variability can markedly influence primer binding efficiency in bisulfite PCR assays, thereby affecting amplification outcomes (Fuso et al. 2015; Candiloro et al. 2017). Furthermore, differences in cellular composition across samples may introduce substantial estimation errors and reduce statistical power for detecting tissue-associated methylation effects. In this study, inter-individual variation in cfDNA methylation signatures was observed for all selected tissue-associated genes except *UPK2*; however, these variations were not significantly altered by treatment. The observed heterogeneity in methylation profiles may therefore influence the interpretation and reproducibility of DNA methylation analyses.

In addition to fragmentation and methylation variability, infectious complications were evaluated as potential confounding factors. Microbial infections are known to induce distinct DNA methylation changes in host cells (Bobetsis et al. 2007; Sankar et al. 2022; Fol et al. 2020), and infections such as cystitis and rectitis are common complications following prostate radiotherapy (Reynaud et al. 2023). However, in our study, no significant or notable changes were detected in the methylation profiles of genes associated with the bladder or rectum in patients who developed infections after radiotherapy. Furthermore, comparisons between patients with and without rectitis before and after radiotherapy did not reveal significant differences in the relative DNA levels of *MUC2* and *MS4A12*. Nevertheless, analyses indicated an increase in *MUC2* levels and a decrease in *MS4A12* levels following radiotherapy in both patient groups. These observed changes may reflect the biological alterations occurring in tissue in response to radiotherapy. Additionally, the increased presence of short DNA fragments could have masked potential infection-related differences.

## Conclusion

This study demonstrates that radiotherapy is associated with a marked increase in cfDNA fragmentation. However, PCR-based analyses reveal that this increase does not correspond to elevated relative DNA levels of tissue-associated genes in serum. The enrichment of short DNA fragments, together with pronounced inter-individual variability in tissue-associated DNA methylation profiles, may weaken the expected association between treatment-induced tissue damage and relative DNA abundance. This suggests that tissue-associated DNA methylation signatures in cfDNA have important methodological and biological limitations when used as independent biomarkers of radiotherapy-induced tissue damage or individual radiosensitivity. Overall, enhancing the effectiveness of methylation-based cfDNA analyses in radiotherapy requires the development of optimized, integrative analytical approaches that account for cfDNA fragmentation patterns and methylation variability.

## Supplementary Information

Below is the link to the electronic supplementary material.

Supplementary Material 1

## Data Availability

All data included in this study are available upon request by contact with the corresponding author.
