# Correction: Assessment of radiotherapy effect and toxicity using tissue-associated DNA methylation markers in cell-free DNA: a study on prostate cancer

**DOI:** 10.1007/s00411-026-01230-x

**Published:** 2026-06-24

**Authors:** Nurten Bahtiyar, Ozlem Mermut, Sinem Firtina, Ahmet Ozaydin, Aisha Suleymanova, Begum Isikgil, İlhan Onaran

**Affiliations:** 1 Department of Biophysics, Cerrahpaşa Faculty of Medicine, Istanbul University-Cerrahpaşa, Kocamustafapaşa Street, Istanbul, 34098 Turkey; 2 Department of Radiation Oncology, University of Health Sciences, Istanbul Training and Research Hospital, Istanbul, Turkey; 3 Department of Medical Genetics, Cerrahpasa Faculty of Medicine, Istanbul University-Cerrahpasa, Istanbul, Turkey; 4 Department of Medical Biology, Cerrahpasa Faculty of Medicine, Istanbul University-Cerrahpasa, Istanbul, Turkey; 5 Department of Medical Biology and Genetics, Institute for Graduate Education, Istinye University, Istanbul, Turkey

**Correction to: Radiation and Environmental Biophysics (2026) 65:461–472**

**https://doi.org/10.1007/s00411-026-01207-w**

In the original version of this article, Begüm Işıkgil at affiliation 'Department of Medical Biology and Genetics, Istinye University, Institute for Graduate Education, Istanbul, Turkey' was missing and İlhan Onaran at affiliation 'Department of Medical Biology, Istanbul University-Cerrahpasa, Cerrahpasa Faculty of Medicine, Istanbul, Turkey' was missing from the author list.

The original article has been corrected.

