# Supplementary material for: Assessment of radiotherapy effect and toxicity using tissue-associated DNA methylation markers in cell-free DNA: a study on prostate cancer
Source: Radiat Environ Biophys. 2026 Mar 6;65(1):461–72. doi: 10.1007/s00411-026-01207-w (PMC13135590; doi:10.1007/s00411-026-01207-w)
Supplement: Supplementary file 1 — Supplementary Material 1 [file 411_2026_1207_MOESM1_ESM.docx]

**Supplementary Table 1.** Tissue-associated genes primers

| **Gene Name** | **Amplicon**  **Bp** | **Amplicon**  **CpG**  **Count** | **Forward Primer**  **(5’-3’)** | **Reverse Primer**  **(5’-3’)** |
| --- | --- | --- | --- | --- |
| **KLK3** | 299 | 4 | GTTTAGGTTATATGGGGAGGTAGG | AAAAAACACAAATAAAACAAAAACC |
| **TGM4** | 233 | 19 | TTTTAGGTTGGAGGGTGGAG | CCTAACCTATAATAAATTTATACTCTAAAA |
| **MUC2** | 137 | 9 | GTTGGTGGAGGAGATTAGTTTTTT | TCTTAATCAACACCTCCTAAATCTC |
| **MS4A12** | 258 | 16 | TGTATTAAGTTATAATAGAAATTTATGTTT | ATCCTAACTAACAAAATAAAACCCC |
| **DHRS2** | 180 | 10 | TTAGAGTTGGGTAGAGGAAGGATAGT | CTCCCCCTACAACTTAACCATAAC |
| **UPK2** | 253 | 27 | TTTGTTTGTTTGTTTGTTTTTTTT | CCAATAACTAAACATACCCC |

**Supplementary Table 2.** Methylation profile of prostate tissue-associated *TGM4* and *KLK3* genes in the patient and control groups

| ***TGM4* gene**  **methylation profile** | **Pre-RT**  **(n=25)** | **Post-RT**  **(n=25)** | **Control**  **(n=27)** |
| --- | --- | --- | --- |
| 100% U | 10 | 13 | 14 |
| 100% H | 8 | 5 | 6 |
| 50% U 50% M | 1 | - | - |
| 50% U 50% H | 3 | 1 | - |
| 50% H 50% M | - | 1 | 3 |
| 25% U 75% H | 1 | - | - |
| 75% U 25% H | - | 4 | 4 |
| 75% H %25 M | 2 | - | - |
| 50% U 25% H 25%M | - | 1 | - |
| ***KLK3* gene**  **methylation profile** | **Pre-RT**  **(n=25)** | **Post-RT**  **(n=25)** | **Control**  **(n=27)** |
| 100% U | 8 | 12 | 15 |
| 100% H | 17 | 13 | 12 |

U: unmethylated; H: hemimethylated; M: methylated**.**

**Supplementary Table 3.** Methylation profile of colon tissue-associated *MS4A12* and *MUC2* genes in the patient and control groups

| ***MS4A12* gene**  **methylation profile** | **Pre-RT**  **(n=25)** | **Post-RT**  **(n=25)** | **Control**  **(n=27)** |
| --- | --- | --- | --- |
| 100% U | 2 | 5 | 14 |
| 100% H | 21 | 20 | 13 |
| 50% U 50% H | 1 | - | - |
| 75% U 25% H | 1 | - | - |
| ***MUC2* gene**  **methylation profile** | **Pre-RT**  **(n=25)** | **Post-RT**  **(n=25)** | **Control**  **(n=27)** |
| 100% U | 14 | 18 | 19 |
| 100% H | 11 | 7 | 8 |

U: unmethylated; H: hemimethylated.

**Supplementary Table 4.** Methylation profile of bladder tissue-associated *DHRS2* and *UPK2* genes in the patient and control groups

| ***DHRS2* gene**  **methylation profile** | **Pre-RT**  **(n=25)** | **Post-RT**  **(n=25)** | **Control**  **(n=27)** |
| --- | --- | --- | --- |
| 100% U | 2 | 4 | 9 |
| 100% M | 11 | 7 | 15 |
| 100% H | - | 2 | - |
| 50% U 50% M | 4 | 7 | 2 |
| 50% U 50% H | - | 2 | - |
| 50% H 50% M | 1 | - | - |
| 25% U 75% M | 2 | 2 | - |
| 75% U 25% M | 5 | 1 | 1 |
| ***UPK2* gene**  **methylation profile** | **Pre-RT**  **(n=25)** | **Post-RT**  **(n=25)** | **Control**  **(n=27)** |
| 100% U | 25 | 25 | 27 |

U: unmethylated; H: hemimethylated; M: methylated**.**

**Supplementary Table 5.** Pairwise Correlations of mtDNA Fragmentation, mtDNA Integrity, Relative DNA Levels, and Methylation Ratios in Pre-Radiotherapy Group (r values)

|  | **mtDNA-79 (2^-∆Ct^)** | **mtDNA-230 (2^-∆Ct^)** | **mtDNA Integrity** | **TGM4 (2^-∆Ct^)** | **KLK3 (2^-∆Ct^)** | **MS4A12 (2^-∆Ct^)** | **MUC2 (methylation ratio)** |
| --- | --- | --- | --- | --- | --- | --- | --- |
| **mtDNA Integrity** | -0.655 | 0.732 | 1 |  |  |  |  |
| **TGM4 (2^-∆Ct^)** | - | 0.583 | 0.627 | 1 |  |  |  |
| **KLK3 (2^-∆Ct^)** | - | 0.794 | 0.511 | - | 1 |  |  |
| **MS4A12 (2^-∆Ct^)** | - | 0.628 | 0.535 | 0.745 | 0.549 | 1 |  |
| **MUC2 (2^-∆Ct^)** | - | - | 0.545 | 0.697 | 0.540 | 0.698 | 1 |
| **DHRS2 (2^-∆Ct^)** | - | - | 0.449 | 0.800 | 0.452 | 0.829 | - |
| **UPK2 (2^-∆Ct^)** | - | - | 0.614 | - | 0.704 | 0.445 | - |
| **TGM4 (methylation ratio)** | - | - | - | - | -0.673 | - | - |
| **DHRS2 (methylation ratio)** | - | - | - | - | - | - | 0.426 |

Data were analyzed using the Spearman’s rank correlation test.

**Supplementary Table 6.** Pairwise Correlations of mtDNA Fragmentation, mtDNA Integrity, Relative DNA Levels, and Methylation Ratios in Post-Radiotherapy Group (r values)

|  | **mtDNA-230 (2^-∆Ct^)** | **TGM4 (2^-∆Ct^)** | **KLK3 (2^-∆Ct^)** | **MS4A12 (2^-∆Ct^)** | **MUC2 (2^-∆Ct^)** | **DHRS2 (2^-∆Ct^)** | **MUC2 (methylation ratio)** |
| --- | --- | --- | --- | --- | --- | --- | --- |
| **TGM4 (2^-∆Ct^)** | 0.635 | 1 |  |  |  |  |  |
| **MS4A12 (2^-∆Ct^)** | - | 0.770 | 0.808 | 1 |  |  |  |
| **MUC2 (2^-∆Ct^)** | - | 0.763 | 0.791 | 0.792 | 1 |  |  |
| **DHRS2 (2^-∆Ct^)** | - | 0.803 | 0.677 | 0.877 | 0.832 | 1 |  |
| **UPK2 (2^-∆Ct^)** | - | - | 0.450 | - | 0.566 | 0.503 | - |
| **MS4A12 (methylation ratio)** | - | - | 0.464 | 0.461 | - | - | - |
| **DHRS2 (methylation ratio)** | - | - | - | - | - | - | 0.454 |

Data were analyzed using the Spearman’s rank correlation test.
